# Noninvasive serum markers for predicting significant liver histopathology in HBeAg-negative chronic HBV-infected patients with normal alanine aminotransferase

**DOI:** 10.1128/spectrum.03941-23

**Published:** 2024-03-01

**Authors:** Yuhua Gao, Mingyang Wang, Xia’nan Liu

**Affiliations:** 1 Department of Clinical Laboratory, The First Affiliated Hospital of Zhengzhou University, Zhengzhou, Henan, China; 2 Key Clinical Laboratory of Henan Province, Zhengzhou, Henan, China; Children's National Hospital, George Washington University, Washington, USA

**Keywords:** HBeAg negative, liver biopsy, histopathology assessment, normal ALT, antiviral treatment

## Abstract

This study is to explore the proportion of significant liver histopathology in hepatitis B e antigen (HBeAg)-negative chronic hepatitis B virus (HBV)-infected patients with normal alanine aminotransferase (ALT) and investigate noninvasive indicators for predicting significant liver histopathology. A total of 201 HBeAg-negative chronic HBV-infected patients with normal ALT who underwent liver biopsy were involved in this study. Significant liver histological changes were defined as necroinflammation grade ≥2 (G ≥ 2) and/or fibrosis stage ≥2 (S ≥ 2). The results showed that 42.3% (85/201) and 45.8% (92/201) of the HBeAg-negative patients with normal ALT have significant liver necroinflammation (G ≥ 2) and fibrosis (S ≥ 2), respectively. High normal ALT (>22 U/L), high level of serum HBV DNA (>3.42 log IU/mL), and low level of prealbumin (PA) (<170 mg/L) were independent predictors for significant liver necroinflammation, and the predictive value of the combined indicators was 0.750 (*P* < 0.001), while high normal ALT (>24 U/L) and high level of FIB-4 (>1.53) were independent predictors for significant liver fibrosis, and the predictive value of the combined indicators was 0.740 (*P* < 0.001). In conclusion, more than 40% of HBeAg-negative patients with normal ALT have significant liver histopathology and require immediate antiviral treatment. ALT, PA, HBV DNA, and FIB-4 can independently predict significant liver inflammation and fibrosis for HBeAg-negative patients with normal ALT. Lowering the treatment threshold of ALT may benefit the HBeAg-negative chronic HBV-infected patients.

**IMPORTANCE:**

Hepatitis B e antigen (HBeAg)-negative chronic hepatitis B virus (HBV)-infected patients with normal alanine aminotransferase (ALT) were supposed to have a low risk of progression to cirrhosis or hepatocellular carcinoma, and it was recommended to regularly follow up or undergo liver biopsy to assess liver histopathology according to the major international guidelines. However, this study indicates that a considerable number of HBeAg-negative chronic HBV-infected patients with normal ALT have significant liver histopathology and require immediate antiviral treatment. Besides, several clinical commonly used noninvasive indicators were found that can be used to predict significant liver histopathology; thereby liver biopsy might be avoided for HBeAg-negative chronic HBV-infected patients with normal ALT.

## INTRODUCTION

Hepatitis B virus (HBV) is a hepatotropic DNA virus, which causes chronic infection to 240 million people, and about 650,000 people die annually due to chronic hepatitis B (CHB) worldwide (1). The natural history of chronic HBV infection can be classified into five phases: hepatitis B e antigen (HBeAg)-positive chronic infection, HBeAg-positive chronic hepatitis, HBeAg-negative chronic infection, HBeAg-negative chronic hepatitis, and hepatitis B surface antigen (HBsAg)-negative phase (2). HBeAg-negative chronic infection, also known as inactive carriers, refers to HBeAg-negative patients with normal alanine aminotransferase (ALT) and low HBV DNA load (2). According to the major international guidelines such as the American Association for the Study of Liver Diseases (AASLD), the European Association for the Study of the Liver (EASL), and the Asian Pacific Association for the Study of the Liver (APASL) (2–4), the indications for antiviral therapy are all targeted at patients with persistent viral replication, abnormal ALT levels exceeding two times, or significant liver histopathology. For HBeAg-negative patients with chronic HBV infection and normal ALT, it is recommended to regularly follow up or undergo liver biopsy to assess liver histopathology (2–4).

However, recent studies reported that a considerable proportion of HBeAg-negative patients with normal ALT showed significant liver histopathology (5–8). A study from Duan et al. (7) suggested that more than 37% of CHB patients with HBeAg-negative and normal ALT have significant liver inflammation or fibrosis, while this proportion can be as high as 67% in another study reported by Wang et al. (5). Therefore, only using ALT to determine the degree of liver inflammation and initiate antiviral treatment might misclassify a fair proportion of patients with significant liver histopathology. Indeed, in clinical practice, it is currently a crucial issue to determine the stage of liver biopsy and accurately identify true “inactive carriers.” Although liver biopsy is considered as the gold standard to assess liver histopathology, it is not practical to use regularly in the clinical management (9). Therefore, it is particularly important to explore noninvasive indicators to predict liver histopathology.

To improve the current clinical management of HBeAg-negative patients with normal ALT, this study involved 201 HBeAg-negative patients with normal ALT who have undergone liver biopsy and investigated the correlation between liver histopathology and clinical commonly used noninvasive serum indicators.

## MATERIALS AND METHODS

### Patients

We retrospectively analyzed the patients who underwent liver biopsy from January 2019 to December 2022 in our hospital. The inclusion criteria were as follows: 18–60 years old, positive HBsAg for at least 6 months, HBeAg-negative, and normal ALT (≤40 U/L for both males and females). The exclusion criteria were as follows: had received HBV antiviral therapy; coinfected with other viruses like hepatitis C virus, hepatitis D virus, hepatitis E virus, or human immunodeficiency virus; and had liver damage induced by other causes (autoimmune hepatitis, nonalcoholic fatty liver disease, Wilson disease, etc.). Finally, a total of 201 patients met our criteria and were included in this study. This study was approved by the ethics committee of The First Affiliated Hospital of Zhengzhou University and conducted in accordance with the ethical standards of the Helsinki Declaration. Informed consent was obtained from all patients.

### Liver biopsy

Liver biopsies were performed for histopathology assessment. The necroinflammation scores and fibrosis scores of the liver tissues were evaluated by experienced pathologists according to the Chinese guidelines for the prevention and treatment of CHB (10). The Scheuer scoring system was adopted to evaluate liver necroinflammation and fibrosis. Briefly, grade of liver necroinflammation ranges from G0 to G4, and liver fibrosis stage was classified into S0–S4. Significant liver histological changes were defined as necroinflammation grade ≥2 (G ≥ 2) and/or fibrosis stage ≥2 (S ≥ 2).

### Laboratory tests

Laboratory test results were extracted from the electronic medical record. HBV DNA was quantified by Roche Cobas TaqMan HBV test (Roche Diagnostics, Mannheim, Germany) with the lower detection limit of 20 IU/mL. Serum biomarkers including ALT, aspartate aminotransferase (AST), γ-glutamyl transpeptidase (GGT), alkaline phosphatase (ALP), total protein (TP), albumin (ALB), prealbumin (PA), total bilirubin (TBIL), direct bilirubin (BILD), and total bile acid (TBA) were detected by Roche Cobas c701 system (Roche Diagnostics, Mannheim, Germany). The upper limit of normal (ULN) of ALT was 40 U/L for both males and females. Platelet was detected by Coulter Cellular Analysis System (Beckman, California, USA). International normalized ratio (INR) and fibrinogen (Fib) were detected by ACL TOP System (Instrumentation Laboratory, Milan, Italy). All the tests were completed in our hospital.

### Formulas

The formulas for AST to platelet ratio (APRI), fibrosis-4 (FIB-4), GGT to platelet ratio (GPR), and GGT to ALB ratio (GAR) were as follows:


$$APRI=(AST/[ULN]/platelet\;[10^{9}/L])\times 100,$$

$$FIB-4=(age[year]\times AST[U/L])/\left\{(platelet\;[10^{9}/L])\times ALT[U/L{]}^{1/2}\right\},$$

GPR=(GGT/ULN)×100/platelet,

GAR=GGT/ALB.

### Statistical analysis

Continuous variables were expressed as means ± standard deviation and were compared using the Student’s *t* test or Mann-Whitney *U* test. Stepwise logistic regression analysis was performed to identify independent predictors for the significant liver histological changes. Receiver operating characteristic (ROC) curve was generated to evaluate the predictive ability of the independent predictors (11). The best cut-off value of the predictor was set up in the condition of the highest Youden index (the sum of sensitivity and specificity minus 1). All statistical analyses were done by the use of SPSS 19.0 (SPSS, Chicago, IL, USA). All statistical analyses were two-tailed, and *P* < 0.05 was considered as statistically significant.

## RESULTS

### Patient characteristics

A total of 201 HBeAg-negative CHB patients (114 males and 87 females) with normal ALT were finally included in this study. The baseline characteristics of the study patients are shown in Table 1. At baseline, 42.3% (85/201) and 45.8% (92/201) of the patients have significant liver necroinflammation (G ≥ 2) and fibrosis (S ≥ 2), respectively. Compared with the patients with none or mild liver histological changes, patients with significant liver histological changes (G ≥ 2 or S ≥ 2) showed significantly higher level of age, INR, ALT, AST, TBA, APRI, FIB-4, and GPR, as well as significantly lower level of PA (all *P* < 0.05). In addition, serum HBV DNA was significantly higher in patients with significant liver necroinflammation compared with patients with none or mild liver necroinflammation (3.55 ± 1.09 vs 3.13 ± 0.86 log IU/mL, *P* = 0.002).

**TABLE 1:** Characteristic of the 201 HBeAg-negative chronic HBV-infected patients with normal ALT

Characteristic	Total	G < 2	G ≥ 2	*P*	S < 2	S ≥ 2	*P*
No. of cases	201	116	85	–^*a*^	109	92	–
Males/females	114/87	62/54	52/33	0.275	61/48	53/39	0.815
Age (years)	44.6 ± 10.6	43.2 ± 11.1	46.4 ± 9.7	0.033	42.3 ± 10.3	47.3 ± 10.4	0.001
HBsAg (log IU/mL)	2.69 ± 0.76	2.67 ± 0.78	2.70 ± 0.74	0.814	2.60 ± 0.79	2.78 ± 0.73	0.095
HBV DNA (log IU/mL)	3.30 ± 0.98	3.13 ± 0.86	3.55 ± 1.09	0.002	3.20 ± 0.83	3.43 ± 1.13	0.099
INR	1.05 ± 0.14	1.03 ± 0.14	1.07 ± 0.14	0.037	1.03 ± 0.15	1.07 ± 0.14	0.026
Fib (g/L)	2.46 ± 0.85	2.50 ± 0.66	2.41 ± 1.05	0.455	2.44 ± 0.65	2.49 ± 1.03	0.638
ALT (U/L)	24.9 ± 8.9	22.6 ± 9.3	28.1 ± 7.5	0.001	22.3 ± 8.9	28.0 ± 7.9	0.001
AST (U/L)	28.6 ± 12.1	26.7 ± 12.5	31.2 ± 11.2	0.01	26.1 ± 12.4	31.6 ± 11.1	0.001
GGT (U/L)	63.7 ± 55.9	55.7 ± 55.7	74.7 ± 54.5	0.017	58.5 ± 58.5	69.8 ± 52.2	0.156
ALP (U/L)	101.2 ± 64.3	94.5 ± 58.6	110.3 ± 70.7	0.086	95.2 ± 62.3	108.4 ± 66.3	0.148
TP (g/L)	66.5 ± 6.2	66.8 ± 6.4	66.1 ± 6.0	0.433	67.1 ± 6.4	65.8 ± 5.9	0.131
ALB (g/L)	39.4 ± 4.8	40.1 ± 4.8	38.5 ± 4.8	0.022	39.9 ± 4.9	38.8 ± 4.7	0.11
PA (mg/L)	180.0 ± 66.1	192.5 ± 64.3	162.8 ± 64.9	0.002	189.3 ± 63.6	168.9 ± 67.6	0.029
TBIL (μmol/L)	16.4 ± 15.1	14.2 ± 11.9	19.4 ± 18.2	0.015	14.5 ± 12.3	18.6 ± 17.7	0.052
BILD (μmol/L)	8.4 ± 9.5	6.9 ± 7.5	10.4 ± 11.4	0.016	7.2 ± 7.7	9.8 ± 11.1	0.058
TBA (μmol/L)	13.4 ± 16.8	10.5 ± 10.8	17.5 ± 21.9	0.007	10.5 ± 9.3	17.0 ± 22.3	0.01
APRI	0.54 ± 0.42	0.45 ± 0.36	0.64 ± 0.47	0.003	0.42 ± 0.33	0.67 ± 0.48	0.001
FIB-4	2.06 ± 1.83	1.79 ± 1.55	2.42 ± 2.10	0.022	1.66 ± 1.51	2.54 ± 2.05	0.001
GPR	0.81 ± 0.92	0.66 ± 0.85	1.01 ± 0.97	0.007	0.68 ± 0.88	0.97 ± 0.94	0.026
GAR	1.70 ± 1.61	1.47 ± 1.61	2.01 ± 1.56	0.017	1.58 ± 1.75	1.85 ± 1.43	0.239

^*a*^ "–" represents not applicable.

### Factors associated with significant liver histological changes

In order to find the independent predictors for significant liver histological changes, we conducted univariate logistic regression analysis, and the indicators with significant differences were then selected for multivariate regression analysis (Table 2). Univariate logistic regression analysis showed that age, HBV DNA, INR, ALT, AST, GGT, ALB, PA, TBIL, BILD, TBA, APRI, FIB-4, GPR, and GAR were associated with significant liver necroinflammation (G ≥ 2), while age, INR, ALT, AST, PA, TBA, APRI, FIB-4, and GPR were associated with significant liver fibrosis (S ≥ 2). Further multivariate regression analysis indicated that HBV DNA, ALT, and PA were the independent predictors for significant liver necroinflammation (G ≥ 2), while ALT and FIB-4 were the independent predictors for significant liver fibrosis (S ≥ 2). Based on this, we derived predictive formulas for significant liver necroinflammation (G ≥ 2) and fibrosis (S ≥ 2), respectively. The formulas were as follows:


Logit P=0.073×ALT−0.008×PA+0.519×HBVDNA−2.541(forG≥2),

**TABLE 2:** Factors associated with significant liver histological changes

Factors	G ≥ 2				S ≥ 2			
	Univariate		Multivariate		Univariate		Multivariate	
	OR (95% CI)^*a*^^,^^*b*^	*P*	OR (95% CI)	*P*	OR (95% CI)	*P*	OR (95% CI)	*P*
Males/females	0.73 (0.41–1.29)	0.275			0.94 (0.53–1.64)	0.815		
Age (years)	1.03 (1.00–1.06)	0.035			1.05 (1.02–1.08)	0.001		
HBsAg (log IU/mL)	1.05 (0.72–1.51)	0.813			1.37 (0.95–1.99)	0.096		
HBV DNA (log IU/mL)	1.58 (1.16–2.13)	0.003	1.68 (1.21–2.34)	0.002	1.28 (0.96–1.71)	0.092		
INR	8.33 (1.09–63.47)	0.041			9.76 (1.25–76.32)	0.030		
Fib (g/L)	0.88 (0.62–1.24)	0.456			1.09 (0.78–1.51)	0.625		
ALT (U/L)	1.08 (1.04–1.12)	0.001	1.08 (1.04–1.12)	0.001	1.08 (1.04–1.12	0.001	1.09 (1.05–1.13)	0.001
AST (U/L)	1.03 (1.01–1.06)	0.012			1.04 (1.02–1.07)	0.002		
GGT (U/L)	1.01 (1.00–1.01)	0.020			1.00 (1.00–1.01)	0.159		
ALP (U/L)	1.00 (1.00–1.01)	0.097			1.00 (1.00–1.01)	0.158		
TP (g/L)	0.98 (0.94–1.03)	0.435			0.97 (0.92–1.01)	0.132		
ALB (g/L)	0.93 (0.88–0.99)	0.023			0.95 (0.90–1.01)	0.111		
PA (mg/L)	0.99 (0.99–1.00)	0.002	0.99 (0.99–1.00)	0.002	1.00 (0.99–1.00)	0.031		
TBIL (μmol/L)	1.03 (1.00–1.05)	0.023			1.02 (1.00–1.04)	0.064		
BILD (μmol/L)	1.04 (1.01–1.08)	0.019			1.03 (1.00–1.07)	0.063		
TBA (μmol/L)	1.03 (1.01–1.06)	0.006			1.03 (1.01–1.06)	0.010		
APRI	2.93 (1.43–6.03)	0.003			4.69 (2.07–10.58)	0.001		
FIB-4	1.21 (1.03–1.42)	0.020			1.33 (1.12–1.59)	0.001	1.40 (1.16–1.69)	0.001
GPR	1.54 (1.11–2.15)	0.011			1.43 (1.03–1.99)	0.031		
GAR	1.24 (1.03–1.49)	0.021			1.11 (0.93–1.32)	0.241		

^*a*^ OR: odds ratio.

^*b*^ CI: confidence interval.


LogitP=0.087×ALT+0.336×(FIB−4)−3.907(forS≥2).

Note: Logit *P*: the probability of significant liver inflammation or fibrosis

### Predictive value of indicators for significant liver histological changes

**Fig 1:**
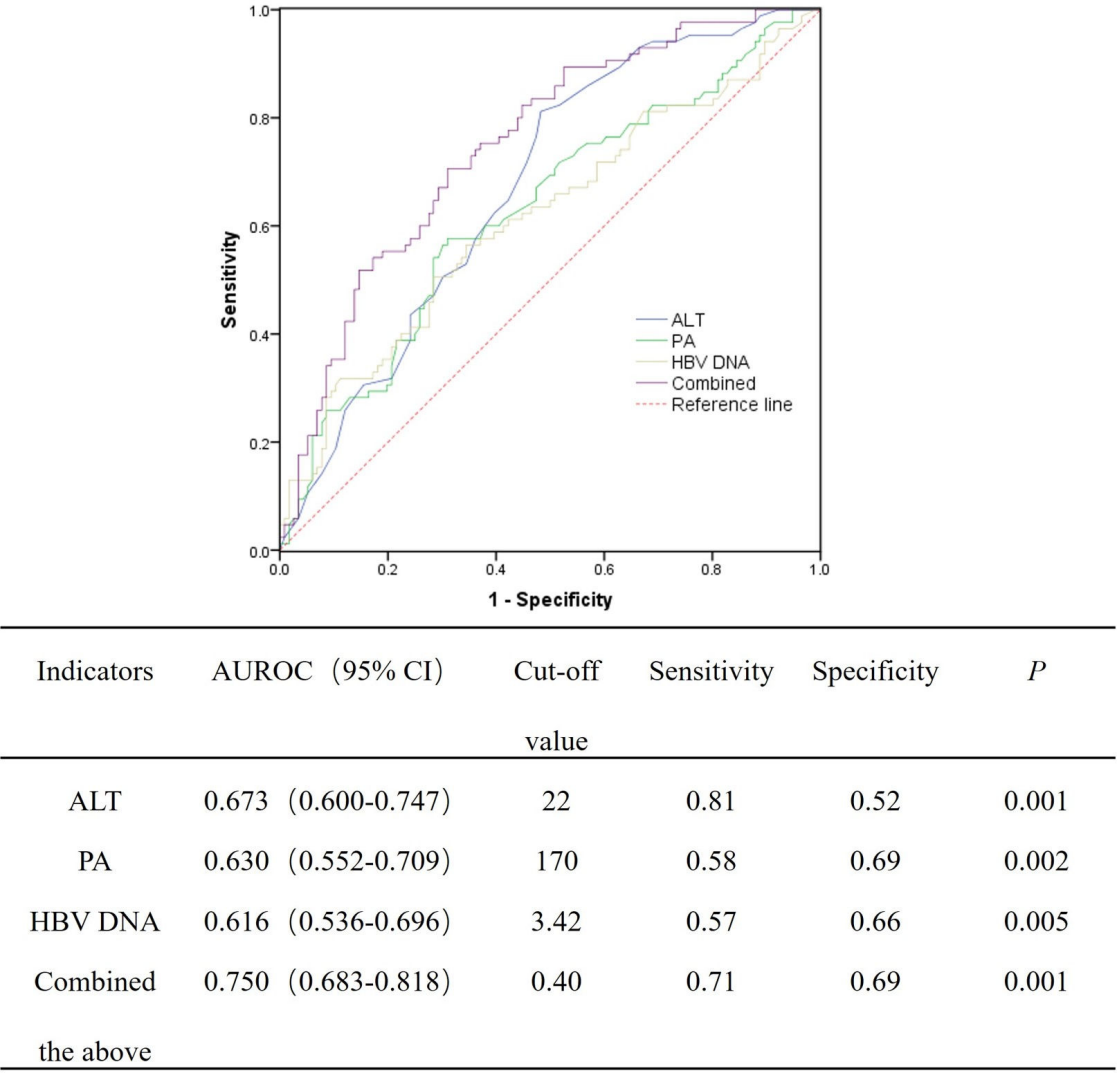
AUROC of the independent indicators for significant liver necroinflammation (G ≥ 2).

**Fig 2:**
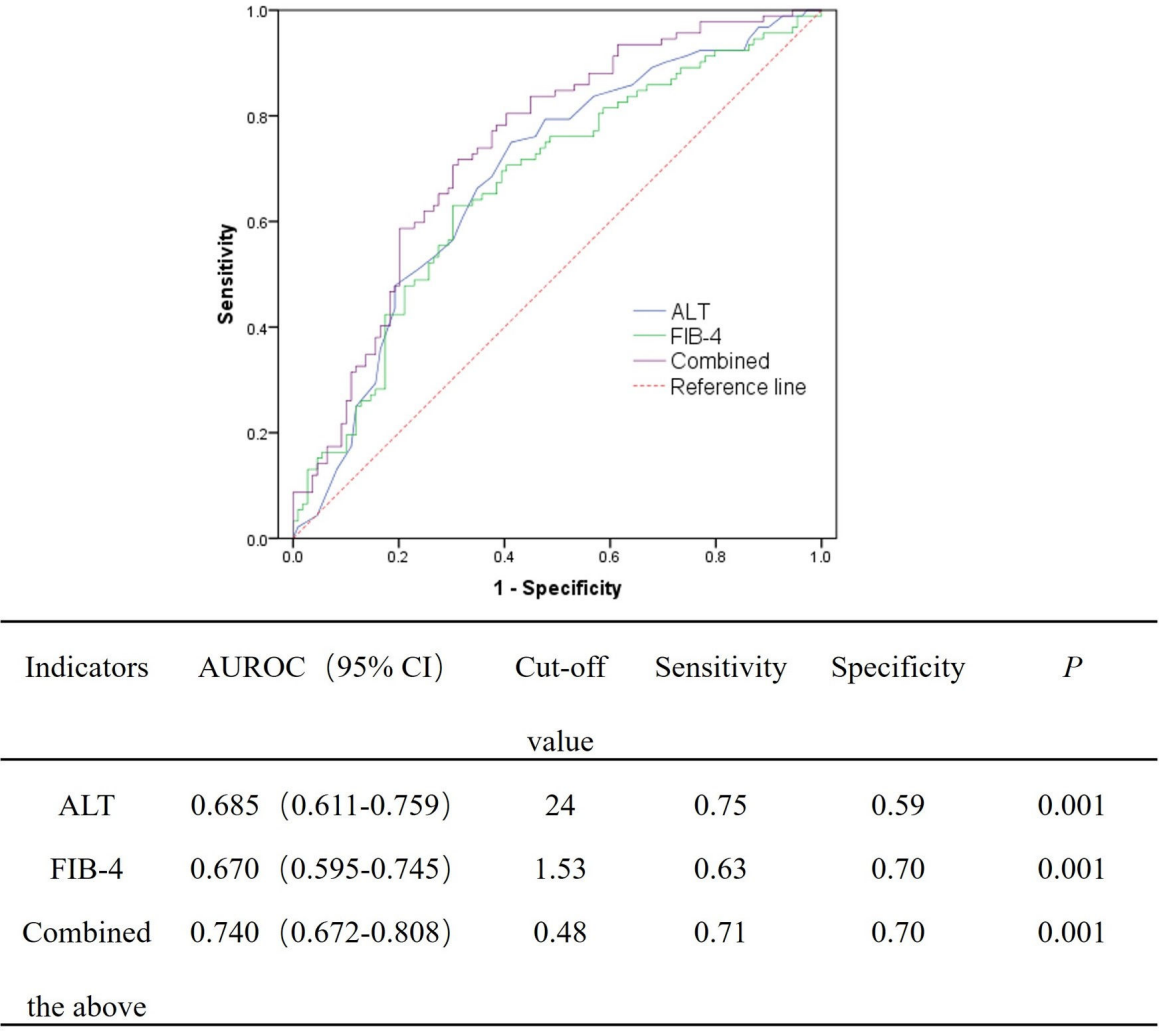
AUROC of the independent indicators for significant liver fibrosis (S ≥ 2).

Furthermore, the predictive value of the independent predictors for significant liver histological changes was then evaluated using ROC curve. The areas under the ROC curve (AUROCs) of ALT, PA, and HBV DNA for predicting significant liver necroinflammation are 0.673, 0.630, and 0.616, respectively. When the above three indicators were combined, the predictive value can be improved to 0.750 (Fig. 1). For predicting significant liver fibrosis, the AUROCs of ALT and FIB-4 were 0.685 and 0.670, respectively. When ALT and FIB-4 were combined, the predictive value can be improved to 0.740 (Fig. 2).

## DISCUSSION

Active chronic liver tissue inflammation is the main cause of liver fibrosis, cirrhosis, and even hepatocellular carcinoma in CHB patients (12). Since the efficacy of antiviral therapy for HBeAg-negative patients with normal ALT is closely related to the degree of liver inflammation and fibrosis, the current guidelines recommend starting antiviral treatment only in patients with chronic HBV infection and signs of continued necroinflammation in the liver (13). However, simple and effective noninvasive methods for assessing liver histopathology of HBeAg-negative patients with normal ALT are still lacking in clinical practice, especially in areas with limited medical resources. In this study, we find that ALT, PA, HBV DNA, and FIB-4 can predict significant liver inflammation and fibrosis for HBeAg-negative CHB patients with normal ALT.

During the natural history of chronic HBV infection, HBeAg-negative conversion is an important point, which implies a reduction or even silencing of viral covalently closed circular DNA transcription activity (14). Patients with HBeAg-negative chronic infection are considered to have a lower risk of disease progression, and liver injury is uncommon (2). However, recent studies reported that a considerable number of HBeAg-negative patients with normal ALT still have significant inflammation or fibrosis (5–8). Duan et al. (7) reported that 37.3% (122/327) and 53.2% (174/327) of HBeAg-negative patients with normal ALT have significant liver necroinflammation and fibrosis, respectively. Yao et al. (15) retrospectively analyzed 117 HBeAg-negative patients with normal ALT and detectable HBV DNA and found that 42.7% (50/117) of them have significant liver inflammation. In this study, we found that the proportions of significant liver necroinflammation and fibrosis in HBeAg-negative patients with normal ALT were 42.3% (85/201) and 45.8% (92/201), respectively, which were consistent with previous studies. At present, it remains controversial whether HBeAg-negative patients with normal ALT should apply antiviral treatment (2–4, 16). However, it is a consensus that patients who exhibit significant liver histopathology require immediate treatment. All the above results indicate that more than 40% of HBeAg-negative patients with normal ALT require prompt antiviral treatment, and ALT is not the only key indicator in determining whether to initiate antiviral therapy for HBeAg-negative CHB patients. The latest CHB guideline of China (2022 version) suggested that CHB patients over 30 years old with normal ALT and positive serum HBV DNA should receive antiviral treatment without considering the pathological changes of liver tissues for the purpose of expanding the treatment population (16). However, the problem of partial response or low-level viremia during treatment may become more prominent due to the inclusion of a significant number of patients with normal or low levels of ALT, and further research is needed on the long-term treatment effects of this population.

Many previous studies have attempted to explore noninvasive indicators for predicting significant liver histopathology in HBeAg-negative chronically infected patients (5, 7, 15, 17, 18). Wang et al. (5) reported that prothrombin time (PT) was an independent risk factor of significant histological disease in the HBeAg-negative patients with normal ALT and high serum HBV DNA load (>2,000 IU/mL). Duan et al. (7) included 327 HBeAg-negative patients with normal ALT (≤40 U/L) and found that patients with high normal ALT (>20 U/L) and high serum HBV DNA load (>2,000 IU/mL) were associated with significant liver histopathology. Besides, Tan et al. (17) found that age was an independent predictor for significant liver histopathology in HBeAg-negative CHB patients with normal ALT. Similar to previous studies, we also found that multiple indicators (including age, ALT, INR, HBV DNA, etc.) were associated with significant liver histopathology in univariate regression analysis. However, multivariate regression analysis indicated that ALT, HBV DNA, and PA are independently associated with significant liver necroinflammation, while ALT and FIB-4 are independently associated with significant liver fibrosis, and age was not an independent predictor for significant liver histopathology. Similar to our results, other studies also reported that age was not an independent predictor for significant liver histopathology (5, 7, 15, 18). The discrepancy might be due to the small sample size in the study conducted by Tan et al., and only 23 patients showed significant liver fibrosis in their study (17). When comparing with HBeAg-positive CHB patients, HBeAg-negative ones showed significantly older age, indicating a long time to obtain HBeAg seroconversion (19). Since this study is focusing on HBeAg-negative population with speculated low disease severity, it also should be noted that complex of HBV mutations existed in CHB patients, which might correlate with disease progression (19, 20). For example, HBV core promoter deletion and/or insertion mutations were found to decrease in parallel with a decline in HBV DNA, HBsAg, ALT, and AST levels and increase in HBeAg loss during antiviral therapy (19).

Duan et al. (7) found a significant correlation between ALT and significant liver histopathology in HBeAg-negative patients with normal ALT, but no ROC curve analysis was further conducted to assess its predictive ability. Zeng et al. (6) reported that the predictive value of ALT for significant liver histopathology was 0.66 in chronic HBV-infected patients, yet they did not combine other indicators for further analysis. In this study, we find that high normal ALT (>22 U/L), high level of serum HBV DNA (>3.42 log IU/mL), and low level of PA (<170 mg/L) mean a high risk of significant liver necroinflammation, and the predictive value of the combined indicators was 0.750 (*P* < 0.001), while high normal ALT (>24 U/L) and high level of FIB-4 (>1.53) mean a high risk of significant liver fibrosis, and the predictive value of the joint indicators was 0.740 (*P* < 0.001). Similar with Duan et al. (7), our study once again demonstrated that high normal ALT levels are associated with significant liver necroinflammation and fibrosis in HBeAg-negative patients with normal ALT. This means that lowering the treatment threshold of ALT may benefit the HBeAg-negative CHB patients. Several previous studies suggested to revise the ULN of ALT (21–23). At present, the ULN of ALT recommended by major international guidelines also vary: the ULN of 40 U/L for both males and females adopted by EASL and APASL (2, 4), the ULN of 35 U/L for males and 25 U/L for females as used in the current AASLD guidelines (3), and the ULN of 30 U/L for males and 19 U/L for females recommended by expert consensus and in the guidelines of AASLD (2016 version), National Institute for Health and Care Excellence (NICE), and East Asia (24–26). The latest CHB guidelines of China also suggested to lower the ULN of ALT (16). However, further studies were needed to establish the threshold of ALT that is suitable for the Chinese population.

Our study has several limitations. First, as a retrospective study, selection bias may be existed. Second, the AUROCs of the biomarkers are all below 0.8, which are not impressive. This phenomenon may be related to the relatively small size of study population and lack of serum HBV RNA, as well as HBV DNA sequencing for genotypes and HBV mutations, which may be potential biomarkers to predict disease severity. Third, as a single-center study, our findings might still require external validation in the future.

In conclusion, our study demonstrated that more than 40% of HBeAg-negative patients with normal ALT have significant liver histopathology and require immediate antiviral treatment. High normal ALT, high level of serum HBV DNA, and low level of PA could predict significant liver necroinflammation, while high normal ALT and high level of FIB-4 could predict significant liver fibrosis. Lowering the treatment threshold of ALT may benefit the HBeAg-negative CHB patients.

## Data Availability

The data presented in this study are included in the article; furher inquiries can be directed to the corresponding author.
